# Remimazolam in pediatric ambulatory anesthesia: a critical review and clinical perspective

**DOI:** 10.3389/fmed.2025.1722706

**Published:** 2025-12-15

**Authors:** Yi Zhang, Hongchen Liu, Linyun Wang, Shuang Guo, Qingjun Zeng, Haishan Cui, Kaiyun Li

**Affiliations:** 1 Department of Anesthesiology, Maternal and Child Health Hospital of Wanzhou District, Chongqing, China; 2 Department of Pediatrics, Maternal and Child Health Hospital of Wanzhou District, Chongqing, China; 3 Nursing Department, Maternal and Child Health Hospital of Wanzhou District, Chongqing, China

**Keywords:** remimazolam, pediatric day surgery, anesthesia, safety, critical appraisal, pharmacokinetics, pharmacodynamics, future perspectives

## Abstract

The management of anesthesia for pediatric day surgery demands rapid onset, precise control, and predictable recovery. Remimazolam, an ultrashort-acting benzodiazepine with esterase-dependent metabolism, theoretically meets these needs. However, current pediatric evidence relies primarily on small observational studies with methodological limitations; data for infants under 2 years and long-term neurodevelopmental outcomes remain virtually absent. We conclude that while remimazolam holds promise, its use must be cautious and restricted to carefully selected scenarios until robust randomized controlled trials become available.

## 1 Introduction

### 1.1 Background and unmet needs

Ambulatory surgery optimizes healthcare resources but demands a “fast-in, fast-out” anesthetic paradigm (1, 2). An ideal regimen must ensure rapid onset and recovery while minimizing adverse events (3, 4). However, current mainstays have limitations: propofol is associated with injection pain, hemodynamic instability, and rare infusion syndromes (5, 6), while sevoflurane carries high risks of emergence delirium (ED) and postoperative nausea and vomiting (PONV) (7–9). Furthermore, traditional benzodiazepines like midazolam suffer from variable hepatic metabolism and active metabolites (10, 11). Remimazolam, with its unique esterase-dependent metabolism, theoretically addresses these challenges.

### 1.2 The need for novel agents, emergence of remimazolam, and critical appraisal of existing literature

Remimazolam, with its unique pharmacological design, may offer a potential option for overcoming some of these limitations. Recent scoping reviews (12, 13) have identified preliminary evidence suggesting acceptable safety and efficacy in pediatric anesthesia, while consistently noting the weak evidence base characterized by small observational studies, inadequate dose exploration, and lack of rigorous RCT designs. Critical knowledge gaps persist, including precise pediatric PK/PD characteristics, validated TCI systems (3), long-term neurodevelopmental safety data, and cost-effectiveness analyses (14–16), particularly for infants under 2 years and children with significant comorbidities (1, 12, 15).

Given the current state of evidence, this review focuses on: (1) elucidating the pharmacological basis of remimazolam, critically examining the quality and strength of evidence supporting its theoretical advantages; (2) evaluating the quality of existing evidence regarding remimazolam’s application across various phases of pediatric day surgery anesthesia, meticulously analyzing design flaws and limitations in interpreting results from available key studies; (3) critically examining its safety profile, particularly risk management strategies for respiratory depression and uncertainties regarding long-term neurodevelopmental outcomes; (4) proposing a framework for individualized clinical decision-making based on comparative assessment of advantages and disadvantages; and (5) identifying the most pressing research deficiencies and suggesting priority directions for future investigation.

### 1.3 Objectives and central thesis

While recent reviews have summarized preliminary pediatric data (12–14), significant gaps remain regarding methodological quality assessment and practical risk management in ambulatory settings. This review distinguishes itself by explicitly linking evidence limitations to a clinical decision-making framework. Our central thesis is that while remimazolam possesses theoretically attractive properties for pediatric ambulatory anesthesia, the current evidence base is insufficient to support routine broad adoption. Its application currently demands rigorous caution, restricted primarily to specific scenarios where its unique profile offers clear benefits over standard agents.

The overarching aim is to provide more cautious decision support for clinical anesthesiologists using remimazolam amidst limited and often flawed clinical evidence, and concurrently, to stimulate further rigorous research in this critical area.

## 2 Methodology: literature search and study selection

This critical narrative review was conducted following a systematic search strategy.

We searched PubMed, Embase, Web of Science, and the Cochrane Library from inception through October 2024. Search terms included combinations of “remimazolam” (or “CNS 7056”) AND “pediatric” (or “children,” “infant”) AND “anesthesia” (or “sedation,” “ambulatory surgery”). Inclusion criteria were: (1) clinical studies involving patients <18 years; (2) use of remimazolam for anesthesia or sedation; and (3) full-text availability in English or Chinese.

We selectively included pivotal adult PK/PD studies and preclinical neurodevelopmental data to provide essential context where pediatric evidence was lacking.

Additionally, we selectively included relevant adult pharmacokinetic and pharmacodynamic studies and preclinical neurodevelopmental safety data where they provided essential context for understanding pediatric applications or highlighting critical knowledge gaps.

Reference lists of identified articles were hand-searched for additional relevant publications.

Given the nascent state of pediatric remimazolam research and the paucity of high-quality randomized controlled trials, this review adopted an inclusive approach prioritizing comprehensive evidence synthesis over strict study quality thresholds, while explicitly appraising methodological limitations throughout.

### 2.1 Molecular mechanism

Remimazolam functions as a positive allosteric modulator of the GABAA receptor (17, 18). Unlike midazolam, it incorporates an ester linkage allowing for rapid hydrolysis (19, 20). Although *in vitro* studies by Kilpatrick (19) suggested slight α1-subunit selectivity, which might imply specific sedative properties, this has not yet translated into clinically distinct pediatric effects compared to other benzodiazepines (18).

### 2.2 Metabolic pathway and organ independence

The ester linkage within remimazolam’s molecular structure is central to its “soft drug” design, allowing rapid hydrolysis by non-specific esterases, primarily carboxylesterase 1 (CES1) present in blood and tissues (predominantly liver), yielding CNS7054, a carboxylic acid metabolite with minimal pharmacological activity that is readily cleared (19, 20).

#### 2.2.1 “Relative” organ independence: pediatric caveats

Drug inactivation occurs independently of hepatic CYP450 enzymes or renal excretion pathways. Adult studies by Stöhr et al. (21) indicated that mild to moderate hepatic impairment (Child-Pugh class A or B) or any degree of renal impairment had limited impact on remimazolam PK. However, in patients with severe hepatic impairment (Child-Pugh class C), clearance was significantly reduced by approximately 40%, and recovery time prolonged by about 60%, suggesting that severely impaired liver function significantly impacts remimazolam clearance likely through reduced esterase activity.

Critically, extrapolating the theoretical advantage of “relative organ independence” to pediatric patients, particularly infants whose CES1 enzyme system is immature, lacks robust evidence and carries potential risks. Hepatic CES1 expression levels and activity in children (especially neonates and infants) are known to be significantly lower than in adults (22, 23), suggesting that the purported advantage of “relative organ independence” may be less pronounced and potentially more variable in this population. Currently, there is a profound lack of high-quality data examining the impact of varying hepatic and renal function statuses across different pediatric age groups (especially those <2 years) on the PK/PD of remimazolam.

#### 2.2.2 Drug interaction risk and individual variability

Reliance on esterase metabolism theoretically reduces CYP450-related PK interactions; however, pharmacodynamic synergy with CNS depressants, especially opioids, remains a primary clinical concern requiring vigilance (19). While esterase hydrolysis should exhibit lower interindividual variability than CYP450 pathways, practical challenges exist: CES1 activity is age-dependent (significantly lower in neonates and infants) (22, 23), genetic polymorphisms affect CES1 expression (22, 23), and certain drugs (e.g., oseltamivir) may inhibit CES1 (22). Specific data linking CES1 activity to remimazolam metabolism across pediatric age groups are unavailable. Consequently, individualized dosing, cautious titration, and close monitoring remain essential.

### 2.3 Pharmacokinetic profile and pediatric discrepancies

#### 2.3.1 Rapid onset: clinical observations and dose-finding limitations

Remimazolam’s appropriate lipophilicity allows rapid blood–brain barrier crossing. Following intravenous administration, sedative and hypnotic effects are typically achieved within a short timeframe (peak effect in 1–2 min in adults (24)). However, studies investigating precise onset time and optimal induction doses in children are very limited and often of low methodological quality. Wu et al. (25), studying preoperative sedation in children aged 1–6 years, reported an ED50 of 0.051 mg/kg (95% CI 0.033–0.065) and ED95 of 0.077 mg/kg (95% CI 0.064–0.161), but this was based on very small sample size (*n* = 23). A review by Tobias (26) summarized pediatric PK models, generally describing a three-compartment model with small volume of distribution (Vd ≈ 0.1 L/kg), consistent with clinically observed rapid onset. Nevertheless, these models are frequently constructed using limited datasets; their stability and predictive accuracy necessitate rigorous validation. The optimal pediatric induction dose remains unclear.

#### 2.3.2 Context-sensitive half-time: adult vs. pediatric discrepancies

The context-sensitive half-time (CSHT), a crucial pharmacokinetic parameter quantifying the time required for plasma drug concentration to decrease by 50% after stopping continuous infusion, differs between adults and children. Schüttler et al. (27), in healthy adult male volunteers, reported a CSHT of approximately 6.8 ± 2.4 min after 4-h continuous infusion, statistically shorter than midazolam (approximately 40 min) and comparable to propofol (approximately 7.5 min).

In contrast, Gao et al. (28) conducted PK modeling analysis in 23 Chinese children aged 2–6 years, predicting a median CSHT of approximately 17 min (IQR: 12–21 min) after 4-h infusion. Critical caveats include: (1) limited sample size (*n* = 23) and narrow age range (2–6 years only) raise concerns about model accuracy and stability; validation in larger studies encompassing broader age range (including infants and neonates) is essential; (2) the pediatric CSHT is markedly longer than adult data, serving as crucial caution against simply extrapolating adult recovery expectations to children, particularly in younger age groups (<2 years) or children with comorbidities; (3) dependence on model predictions means these predicted CSHT values carry substantial uncertainty.

While 17 min classifies as “ultrashort” compared to midazolam, it is notably longer than the adult value (~7 min). In high-volume pediatric ambulatory centers with strict turnover targets (e.g., 15-min intervals), this difference is clinically relevant. It implies that unlike in adults, remimazolam may not offer a throughput advantage over propofol in children, and recovery protocols cannot be simply extrapolated from adult guidelines.

#### 2.3.3 Pediatric PK/PD characteristics and precision dosing challenges

Age-dependent pharmacokinetic and pharmacodynamic characteristics present significant hurdles to achieving individualized precision dosing in children. Li et al. (29), in a prospective dose-finding study involving 52 children aged 3–12 years receiving remimazolam combined with sufentanil for laryngeal mask airway insertion, confirmed this age dependency: ED95 for suppressing response to stimulation was significantly higher in preschool children (3–6 years: 0.554 mg/kg, 95% CI: 0.515–0.688) compared to school-aged children (7–12 years: 0.504 mg/kg, 95% CI: 0.467–0.635). While this study provides valuable dose references for specific age groups and clinical scenarios, its generalizability is limited. The extreme scarcity of high-quality PK/PD data for infants under 2 years of age represents the single largest obstacle to safe application of remimazolam in this vulnerable population and constitutes the most urgent knowledge gap for future research (15, 16). Without reliable data, developing evidence-based dosing regimens and validating TCI systems remain infeasible.

### 2.4 Key pharmacodynamics: theoretical advantages versus pediatric reality

#### 2.4.1 Hemodynamic effects: relative stability vs. clinical reality

Multiple studies suggest that remimazolam may offer relatively better hemodynamic stability compared to propofol, often characterized by higher mean arterial pressure (MAP) and heart rate (HR) (5, 30). Fang et al. (5) conducted a multicenter, randomized, single-blind, controlled trial (RCT) involving 290 healthy children aged 3–6 years (ASA physical status I-II). They compared remimazolam (induction 0.3 mg/kg, maintenance 0.2–1.0 mg/kg/h) with propofol (induction 2.5 mg/kg, maintenance 6–15 mg/kg/h). Results showed that within the first 5 min post-induction, SBP and HR were significantly higher in the remimazolam group. The incidence of bradycardia during induction (HR < 60 bpm or >30% decrease from baseline) was also significantly lower with remimazolam compared to propofol (*p* < 0.001). Intraoperatively, blood pressure remained relatively stable in both groups, with a low and non-significantly different incidence of hypotension (SBP decrease >20% from baseline) (<1% vs. 2%).

While this study holds value due to its RCT design and large sample size, it has several key methodological limitations: (1) Lack of blinding for key investigators: Anesthesiologists administering the drugs were unblinded due to distinct differences in drug appearance and dosages (clear vs. milky white), potentially introducing significant performance bias. (2) Limited depth of anesthesia assessment: Anesthetic depth was guided solely by the subjective Modified Observer’s Assessment of Alertness/Sedation (MOAA/S) scale, lacking objective monitoring (e.g., Bispectral Index [BIS] (67), entropy, or near-infrared spectroscopy (68)). Benzodiazepines, including remimazolam, are known to potentially inflate BIS values at comparable clinical sedation levels (discussed further in Section 3.2), meaning the actual anesthetic depth might have differed between the groups. (3) Homogeneous study population: The study exclusively included relatively healthy children aged 3–6 years (ASA I-II) undergoing minor elective surgeries. These findings cannot be readily extrapolated to children of other ages, those with higher ASA classifications, obese children, or those undergoing complex or prolonged surgical procedures – precisely the populations where hemodynamic stability is often of greatest clinical concern. (4) Potential issues with confounding control: Ensuring complete consistency in fluid management strategies, opioid administration (type, dose, timing), and vasopressor use across multiple centers is challenging within the study protocol, potentially influencing the observed hemodynamic outcomes.

In contrast, a large retrospective cohort analysis by Kimoto et al. (31) involving 418 children reported that 75.2% experienced a fluctuation in mean arterial pressure (MAP) exceeding 20% from baseline, and 49.3% had fluctuations exceeding 30%. Ephedrine intervention was required in 5% of cases. While notable for its large sample size, this study is constrained by: (1) Inherent flaws of retrospective design: Non-standardized data collection introduces risks of recording bias, missing information, and selection bias. (2) Severe confounding factors: Remimazolam was invariably co-administered with other anesthetic agents in highly varied combinations. Consequently, the observed circulatory fluctuations cannot be definitively attributed to remimazolam itself versus the complex interplay of adjunct medications, surgical stimuli, underlying patient conditions, or volume management strategies. (3) Lack of a control group: Without comparison to other anesthetic regimens, it’s impossible to determine if the observed fluctuation frequency differs from standard practice. (4) Arbitrary dose selection: Dosing was often based on adult experience or “clinical judgment,” lacking standardized protocols derived from pediatric PK/PD studies, potentially leading to under- or over-dosing. Therefore, this study primarily reflects the reality that hemodynamic fluctuations are common during the complex clinical use of remimazolam, necessitating vigilance and enhanced monitoring. It neither refutes remimazolam’s potential hemodynamic advantage relative to propofol nor directly attributes the observed volatility solely to remimazolam.

Shimizu et al. (32), in a retrospective study comparing remimazolam (*n* = 56, combined with fentanyl, dexmedetomidine, ketamine) versus sevoflurane (*n* = 56, combined with fentanyl) for anesthesia during pediatric cardiac catheterization, found that the intraoperative SBP-to-baseline ratio was significantly higher in the remimazolam group (91.4% ± 15.2% vs. 83.2% ± 11.4%, *p* = 0.03), suggesting better maintenance of relative blood pressure. However, the incidence of hypotension was not significantly different between groups (39.3% vs. 46.4%, *p* = 0.79). This retrospective study, confounded by polypharmacy, indicates that even if remimazolam indicates superiority over sevoflurane on certain metrics, the risk of clinically significant hypotension remains substantial, particularly in children with fragile circulations due to congenital heart disease.

#### 2.4.2 Virtually no injection pain: a clear clinical advantage

Remimazolam causes virtually no pain upon intravenous injection. This has been consistently confirmed by multiple studies and extensive clinical experience (5, 33). This represents a distinct and significant clinical advantage over agents like propofol, improving the patient experience and compliance during IV induction, thereby reducing crying and agitation.

#### 2.4.3 Reversibility: a safety net requiring cautious application

The effects of remimazolam can be rapidly and effectively reversed by the specific benzodiazepine antagonist, flumazenil (27, 34). Theoretically, given remimazolam’s relatively short elimination half-life and CSHT, the risk of resedation following flumazenil reversal might be lower compared to reversing longer-acting traditional benzodiazepines. This reversibility may provide an additional safety measure for managing overdose, severe respiratory depression, or specific situations requiring urgent neurological assessment (34, 35). However, several important caveats must be emphasized: (1) Routine reversal is unnecessary and generally not recommended. Given remimazolam’s intrinsic rapid clearance and recovery profile, routine antagonism not only adds drug cost and potential adverse effects but may also mask underlying unresolved issues such as pain or agitation. (2) Flumazenil itself can cause side effects, including anxiety, agitation, dizziness, nausea, and vomiting. Particularly in patients on chronic benzodiazepine therapy or those with a history of seizures, rapid reversal can potentially precipitate withdrawal symptoms or seizures. (3) If flumazenil administration is deemed necessary, it must be done cautiously, titrating slowly (adult starting dose typically 0.2 mg; pediatric dose should be weight-based, e.g., 0.01 mg/kg), with close monitoring of vital signs. (4) Extended monitoring post-reversal is mandatory. Even with remimazolam’s relatively short CSHT, the risk of resedation and recurrent respiratory depression can persist under certain circumstances. Therefore, vigilance must be heightened after reversal to ensure patient safety.

## 3 Clinical application of remimazolam in pediatric day surgery anesthesia: evidence analysis and practical considerations

### 3.1 Anesthetic induction: strategies, advantages, and unresolved dosing challenges

#### 3.1.1 Intravenous induction: the core route with dose uncertainty

Intravenous induction offers a pain-free alternative to propofol. Fang et al. (5) reported high induction success rates in preschoolers comparable to propofol, while Li et al. (29) confirmed significant age-dependent dosing requirements (Table 1). However, standardized dosing for infants remains undefined (25, 26).

**Table 1:** Summary and critical appraisal of key evidence for remimazolam: pediatric clinical studies and relevant adult pharmacokinetic data.

Study (reference)	Type of trial	Patients and comparison	Context/Setting	Dose (Remimazolam, if specified)	Main findings	Critique/Limitations (as highlighted in the review text)
Section A: Pediatric clinical studies						
Fang et al. (5)	Multicenter RCT (single-blind)	290 children (3–6 years, ASA I–II); Remimazolam vs. Propofol	GA for minor elective surgery	Induction: 0.3 mg/kg; Maintenance: 0.2–1.0 mg/kg/h	Better initial hemodynamics; significantly lower bradycardia (9.0% vs. 26.2%). Faster recovery times (extubation: 9.0 vs. 11.9 min). No injection pain. Similar induction success/AEs.	Lack of blinding (anesthesiologists) due to drug appearance/dose differences (potential performance bias); Subjective depth assessment (MOAA/S only, no BIS/EEG); Homogeneous population (healthy 3–6 years, ASA I-II, minor surgery limits generalizability); Potential confounding (consistency of fluids, opioids, vasopressors across centers challenging).
Gao et al. (28)	PK modeling study	23 Chinese children (analyzed, 24 enrolled, 2–6 years)	GA	Used to derive PK parameters (CL = 15.9 mL/kg/min, Vc = 0.11 L/kg)	Estimated pediatric CSHT (~17 min, IQR: 12–21 min) significantly longer than adults (~7 min), though still ultrashort-acting for this age group.	Small sample size (*n* = 23); Narrow age range (2–6 years. only); Model limitations (results are predictions with substantial uncertainty, need validation); Cannot simply extrapolate adult recovery expectations to children (underscores potential slower elimination rate).
Long et al. (36)	Dose-finding (biased coin design)	80 children (1–6 years, split *n* = 40 per group: 1–3 and 3–6 years)	Preoperative anxiolysis (Intranasal – IN)	ED95: 1.57 mg/kg (1–3 years), 1.09 mg/kg (3–6 years)	Determined IN ED95 for sedation. Onset ~5 min, peak ~10 min, effect diminished after ~20 min.	BCD design instability (small N per group); Sparse data at intermediate doses, potential overconcentration at higher doses; No covariate adjustment (age vs. weight); Isotonic regression limitations (monotonicity assumption, unreliable extrapolation/cliff effect); Needs larger N, finer doses, control group, alternative models (e.g., Logistic).
Cai et al. (37)	RCT	60 healthy children (3–7 years, ASA I); IN Remimazolam vs. IN Dexmedetomidine	Preoperative anxiolysis (Intranasal – IN)	1.5 mg/kg	IN Remimazolam had faster onset and recovery than IN Dexmedetomidine.	Extremely high nasal irritation (80%) with IN Remimazolam (major barrier to routine use); Potential assessment bias (timing might miss dexmedetomidine peak); Limited population (healthy ASA I only). IN route not ready for routine clinical use until less irritating formulation developed.
Li et al. (29)	Prospective dose-finding	52 children (3–12 years, split 3–6 & 7–12 years)	LMA insertion (with sufentanil)	ED95: 0.554 mg/kg (3–6 years), 0.504 mg/kg (7–12 years)	Confirmed age-dependent dosing (higher ED95 in younger group). Provided dose reference for specific scenario/combination.	Limited generalizability (specific context/combination only); Underscores challenge of individualized dosing; Highlights extreme data scarcity, especially for infants <2 years. (largest obstacle).
Cai et al. (47)	Double-blind RCT	150 children (1–6 years); Remimazolam (infusion or bolus) vs. Placebo	GA (laparoscopic hernia); ED prevention (PAED ≥10)	Infusion: 1 mg/kg/h OR Bolus: 0.2 mg/kg (at end)	Remimazolam significantly reduced ED incidence (from 35% to 5–8%) vs. placebo, without delaying recovery.	Single-center, specific surgery (limits generalizability); No dose–response investigation (optimal/minimal dose unknown); Short-term outcomes only (no long-term neurodevelopmental follow-up).
Yang et al. (48)	RCT	120 children (3–7 years); Remimazolam bolus vs. control.	GA (tonsillectomy); ED prevention (PAED ≥12)	Bolus: 0.2 mg/kg (at end)	Remimazolam bolus significantly reduced ED incidence (from 44 to 12%) vs. control, without delaying recovery or increasing AEs.	Single-center, specific surgery (limits generalizability); No dose–response investigation; Short-term outcomes only (no long-term neurodevelopmental follow-up).
Kimoto et al. (31)	Retrospective cohort analysis.	418 children.	Adjunct to GA (varied combinations).	Varied, non-standardized.	High frequency of MAP fluctuations observed (>20% in 75, >30% in 49%). Ephedrine needed in 5%. Agitation ~1.9%, somnolence ~0.7%.	Retrospective limitations (recording/missing data/selection bias); Severe confounding (polypharmacy, varied strategies); No control group; Arbitrary dosing (based on adult experience/judgment, not pediatric PK/PD); Cannot attribute fluctuations solely to remimazolam; Reflects complex clinical use reality.
Shimizu et al. (32)	Retrospective comparative.	112 children (with CHD); Remimazolam combo vs. Sevoflurane combo.	GA for cardiac catheterization (with polypharmacy).	Combination therapy (details variable).	Higher SBP-to-baseline ratio (better relative BP) with Remimazolam. No significant difference in high incidence of hypotension (~40%) between groups.	Retrospective limitations; Confounded by polypharmacy (highly varied combos); High hypotension risk remains substantial even if relative BP is better, especially in fragile (CHD) patients.
Hosokawa et al. (46)	Retrospective observational study.	39 children (2 months – 16 years) with CHD; No direct comparator group within study.	GA for cardiac catheterization.	Used for maintenance (specific doses not detailed in review text summary).	Median recovery time (infusion stop to extubation) = 15 min (IQR 12–17 min). Noted as faster than an adult study comparison. However, 15% (6/39) required flumazenil due to inadequate recovery (somnolence/resp. compromise).	Retrospective design; Focus on specific high-risk population (CHD); Need for flumazenil in 15% indicates significant individual variability and potential for delayed recovery despite rapid median time; Comparison to adult study is indirect.
Hirano et al. (45)	Retrospective analysis (pilot).	48 children.	Procedural sedation (Imaging/MRI).	Median: 0.17 mg/kg load, 0.8 mg/kg/h; High co-med use (95% ketamine, propofol, fentanyl).	Procedures successful, no serious airway events. Hemodynamic fluctuations common (MAP ≥20% in 88%, HR ≥ 20% in 54%) but did not require intervention.	Retrospective limitations; Small sample size; High heterogeneity of co-medication (limits conclusions on remimazolam alone); Supports potential safety specifically within combination regimens, not necessarily in isolation.
Section B: Adult pharmacokinetic/pharmacodynamic studies informing pediatric context						
Schüttler et al. (27)	PK/PD study (continuous infusion).	Healthy adult male volunteers (*N* = not specified in review excerpt).	GA with continuous infusion for PK/PD characterization.	Continuous infusion (4-h duration studied)	Adult CSHT approximately 6.8 ± 2.4 min after 4-h infusion. Shorter than midazolam (~40 min), comparable to propofol (~7.5 min) but via different metabolic pathway. CSHT remained relatively stable with increasing infusion duration.	Adult population only (Caution: Do not extrapolate directly); Direct extrapolation to pediatric patients, especially infants, requires caution given age-dependent differences in esterase activity, organ maturation, and drug distribution; Provides theoretical pharmacological context but does not validate pediatric safety or efficacy.

Adult pharmacokinetic and pharmacodynamic studies are included in Section B to provide essential pharmacological context given the paucity of pediatric pharmacometric data and the need to understand developmental differences. Direct extrapolation of adult findings to pediatric populations should be approached with caution given known age-dependent differences in drug metabolism, clearance, and organ function maturation. These studies are referenced throughout the manuscript when discussing the theoretical pharmacological basis for remimazolam use, while explicitly noting the limitations of cross-population inference. This table primarily summarizes pediatric clinical studies. The adult study by Schüttler et al. is included solely to provide comparative context regarding context-sensitive half-time, as pediatric data for these parameters are currently limited. PK, Pharmacokinetic; PD, Pharmacodynamic; RCT, Randomized controlled trial; ED, Effective dose; CSHT, Context-sensitive half-time; ED, Emergence delirium; PAED, Pediatric anesthesia emergence delirium scale; PONV, Postoperative nausea and vomiting; ASA, American Society of Anesthesiologists physical status classification; BCD, Biased-coin design; CI, Confidence interval.

#### 3.1.2 Exploration of intranasal administration: theoretical feasibility versus practical obstacles

Intranasal (IN) administration theoretically offers several advantages, including rapid absorption, avoidance of first-pass hepatic metabolism, and obviating the need for IV access, making it an attractive route for exploration, particularly for preoperative anxiolysis. Long et al. (36), using a biased coin up-and-down design in 80 children aged 1–6 years, determined the ED95 of IN remimazolam for achieving satisfactory sedation/anxiolysis: 1.57 mg/kg for children aged 1–3 years and 1.09 mg/kg for those aged 3–6 years, with onset at approximately 5 min, peak effect at approximately 10 min, but effect noticeably diminishing after 20 min. However, this study’s design faces several critiques including dose allocation instability inherent in biased coin design with small sample sizes, potential overconcentration of subjects at higher doses with insufficient data at intermediate doses, failure to account for covariate influence, and limitations of isotonic regression analysis. Future studies should consider larger sample sizes, finer dose gradients, complementary model-based analyses, and inclusion of control groups to enhance robustness.

Cai et al. (37) conducted an RCT comparing IN remimazolam (1.5 mg/kg, *n* = 30) with IN dexmedetomidine (2 mcg/kg, *n* = 30) for preoperative anxiolysis in healthy children aged 3–7 years. Remimazolam demonstrated faster onset (10 vs. 25 min) and more rapid postoperative recovery (emergence time 22 vs. 44.5 min; PACU stay 34.5 vs. 56 min). However, this study was significantly hampered by an extremely high incidence of nasal mucosal irritation: 80% (24/30) of children receiving IN remimazolam exhibited noticeable signs of nasal irritation (e.g., crying, rubbing the nose, sneezing). This high incidence and severity currently represent the most significant barrier to routine clinical application of IN remimazolam. Additional limitations included potential bias in assessment timing and restricted study population (healthy ASA I children).

Clinical recommendation: Given the prohibitively high incidence (80%) of nasal irritation reported in recent trials, intranasal remimazolam in its current formulation is not recommended for routine pediatric practice. It should be considered experimental until reformulated (e.g., buffered solutions) to improve tolerability.

### 3.2 Anesthetic maintenance: continuous infusion, multimodal integration, and significant monitoring challenges

#### 3.2.1 Complexity of dose titration and depth management

Anesthetic maintenance with remimazolam is typically achieved using continuous intravenous infusion (CIVI). Reported CIVI dose ranges in the literature are extremely wide (e.g., 0.2–3 mg/kg/h) (31, 38), strongly indicating substantial interindividual variability and the absence of a standardized maintenance dose. Consequently, highly individualized titration based on continuous assessment of anesthetic depth is imperative. A study by Shirozu et al. (39) in adults found that during remimazolam maintenance anesthesia (infusion rates 0.5–2.0 mg/kg/h), BIS values were relatively high (median range 38.3–72.0) even when clinically adequate surgical anesthesia was achieved. Compared to propofol, the EEG under remimazolam exhibited significantly increased beta wave (13–25 Hz) activity. This EEG characteristic is similar to that observed with other benzodiazepines (e.g., midazolam) and reflects a “class effect.” This finding implies that sole reliance on BIS numerical values to gauge the depth of anesthesia under remimazolam may be misleading. Clinical judgment must integrate clinical signs, response to surgical stimuli, and potentially other sedation scales alongside BIS monitoring, potentially including analysis of raw EEG waveforms or spectral indices. Regarding compatibility with neuromonitoring, a case report by Kondo et al. (40) indicated that remimazolam combined with remifentanil for spinal surgery did not significantly suppress motor evoked potentials (MEPs), suggesting compatibility. Similarly, Kamata et al. (41) reported feasible recording of MEPs with good signal quality via direct cortical stimulation in a 10-year-old child undergoing brain tumor resection under remimazolam anesthesia (0.6–1.0 mg/kg/h).

#### 3.2.2 Absolute necessity of multimodal analgesia and lack of strategic evidence

Remimazolam inherently lacks analgesic properties. Therefore, implementing multimodal analgesia strategies is essential during any procedure involving noxious stimuli. This necessitates the judicious use of opioid analgesics, albeit with heightened vigilance for the potential synergistic risk of respiratory depression (19, 33). A core strategy for achieving “opioid-sparing” or even “opioid-free” anesthesia involves the effective integration of regional anesthesia techniques (42). Rational combination with non-opioid analgesics (e.g., nonsteroidal anti-inflammatory drugs [NSAIDs], ketamine, or esketamine) is also crucial. However, there is currently an extreme paucity of high-quality clinical evidence guiding the optimal integration of various analgesic modalities, including choice of agent, dosage, timing, and combination regimens, specifically within the context of remimazolam-based anesthesia. Existing studies provide only sparse clues (29, 41).

Some preliminary exploratory studies have investigated combination strategies. For instance, Chen et al. (43), in an RCT involving children undergoing fiberoptic bronchoscopy (*n* = 90), found that a combination of remimazolam (0.15 mg/kg load + 0.5–1 mg/kg/h maintenance) and low-dose propofol (1 mg/kg/h) significantly reduced the incidence of respiratory depression (combination group 6.7% vs. remimazolam alone 20% vs. propofol alone 23.3%) while maintaining similar sedation effectiveness. The study by Li et al. (29) explored induction doses of remimazolam combined with sufentanil for LMA insertion. In clinical practice, anesthesiologists must design individualized balanced anesthesia and analgesia plans incorporating remimazolam, tailored to the surgical type, patient characteristics, and anticipated pain level, while acknowledging the current insufficiency of evidence regarding optimal combination regimens. Future studies should focus on defining optimal dosing strategies for specific drug combinations to maximize synergy and minimize adverse effects.

#### 3.2.3 Coexisting potential and limitations in specific scenarios (procedural sedation)

For non-surgical procedures requiring moderate to deep procedural sedation, such as gastrointestinal endoscopy (33, 43, 44), diagnostic imaging (e.g., MRI) (45), or cardiac catheterization (32), remimazolam’s pharmacological profile (rapid onset, controllability, relative safety) might offer distinct advantages. Nevertheless, safety considerations, particularly the ever-present risk of respiratory depression, remain the foremost concern. Hirano et al. (45) retrospectively analyzed 48 children who received remimazolam (median dose 0.17 mg/kg load, 0.8 mg/kg/h maintenance) for sedation during imaging studies (primarily MRI). They found that all procedures were successfully completed without any serious adverse events requiring airway intervention. However, hemodynamic fluctuations were notably common (MAP changes ≥20% in 88.4% of patients, ≥30% in 62.8%; HR changes ≥20% in 54.3%), although none required pharmacological intervention. Furthermore, a high proportion of patients (95%) received concomitant sedative agents (ketamine, propofol, or fentanyl). While these findings support the potential safety profile of remimazolam within combination regimens, especially for potentially longer procedural sedation tasks, the study’s retrospective design, small sample size, and the heterogeneity of co-medication protocols significantly limit the reliability and generalizability of its conclusions.

### 3.3 Recovery dynamics and quality

#### 3.3.1 Recovery dynamics: theoretical rapidity versus practical uncertainties

Remimazolam demonstrates rapid recovery dynamics. Fang et al. (5) observed significantly shorter extubation and discharge times compared to propofol, and Hosokawa et al. (46) noted rapid wake-up times in cardiac catheterization (Table 1). However, PK modeling suggests the pediatric context-sensitive half-time is notably longer than in adults (27, 28), cautioning against direct extrapolation of adult turnover expectations.

#### 3.3.2 Improving recovery quality: potential for ED reduction and evidence limitations

Emergence delirium (ED) is a frequent and distressing complication following pediatric anesthesia, particularly prevalent in preschool-aged children and notably associated with the use of sevoflurane (7, 8). Initial studies suggest remimazolam might help reduce the incidence of emergence delirium. Cai et al. (47), in a double-blind RCT involving 150 children aged 1–6 years undergoing laparoscopic hernia repair, compared three interventions’ effects on ED (defined as PAED scale score ≥10). Compared to a 35% ED incidence in the placebo group, both continuous infusion of remimazolam (1 mg/kg/h) and a single bolus dose (0.2 mg/kg) at the end of surgery significantly reduced ED incidence to 5% (*p* = 0.001) and 7.7% (*p* = 0.003), respectively. Yang et al. (48), in another RCT studying 120 children aged 3–7 years undergoing tonsillectomy, observed similar results. A single end-of-procedure bolus of remimazolam (0.2 mg/kg) significantly reduced the incidence of ED (defined as PAED score ≥12) from 44% in the control group to 12% (*p* < 0.001). Importantly, both studies indicated that this benefit in reducing ED did not lead to delayed recovery or an increase in other adverse events.

However, these studies also possess critical limitations: (1) They were predominantly single-center studies focused on specific surgical types, which limits the generalizability of the findings. (2) They lacked investigation into the dose–response relationship, failing to identify the optimal or minimal effective dose for ED prevention. (3) The focus was solely on short-term outcomes, with no long-term follow-up assessments of potential impacts on neurocognitive function or other developmental aspects, a crucial gap identified in the safety assessment.

#### 3.3.3 Impact on PONV: theoretical rationale versus pediatric evidence vacuum

Volatile inhaled anesthetics, such as sevoflurane, are established independent risk factors for postoperative nausea and vomiting (PONV). Theoretically, employing remimazolam within a total intravenous anesthesia (TIVA) technique should help reduce the incidence of PONV by avoiding volatile agents. Yoo et al. (49), in a small RCT involving adult women undergoing gynecologic laparoscopic surgery, found that the remimazolam group experienced significantly lower PONV incidence over 24 h postoperatively (5% vs. 45%, *p* = 0.003) and required significantly fewer rescue antiemetics (0% vs. 30%, *p* = 0.020) compared to the sevoflurane group. While this study provides preliminary suggestive evidence for an anti-PONV advantage of remimazolam-based TIVA, its limitations are considerable: (1) The study population consisted of adults (specifically women undergoing gynecologic laparoscopy, a group inherently at high risk for PONV), whose physiological characteristics and PONV risk factors differ substantially from those of children. (2) It was a single-center study with an extremely small sample size (*n* = 40), compromising the stability and reliability of the conclusions. (3) Significant confounding factors were present and potentially uncontrolled, including widespread use of prophylactic antiemetics (ramosetron), routine administration of flumazenil for reversal, and potential differences in pain management and volume status between groups, all of which obscure the independent effect of remimazolam on PONV.

As emphasized in the fourth consensus guidelines for PONV management by Gan et al. (50), PONV is a multifactorial problem. The optimal management strategy involves risk stratification followed by multimodal prevention, including appropriate anesthetic choices (favoring TIVA over volatile agents in high-risk patients), avoidance of other high-risk drugs (like nitrous oxide or etomidate), and stepwise prophylactic antiemetic therapy (e.g., using 5-HT₃ receptor antagonists, dexamethasone, droperidol/haloperidol, aprepitant). Therefore, even if remimazolam indicates potential in reducing PONV, large-scale, multicenter RCTs specifically targeting pediatric populations are urgently needed (evidence gaps detailed in Section 1.3). These future studies must not only optimize dosing regimens and assess long-term safety but also clearly define the precise role and effectiveness of remimazolam within the framework of multimodal PONV prevention strategies tailored for children.

## 4 Safety profile and risk management

### 4.1 Core safety profile: respiratory and hemodynamic stability

Respiratory depression remains the primary risk, necessitating strict titration and routine ETCO2 monitoring (16, 20, 51). While comparative data suggest remimazolam may cause less frequent respiratory events than propofol (33, 52), vigilance is mandatory, especially in high-risk cohorts (38). Regarding hemodynamics, RCTs indicate remimazolam offers better stability with less bradycardia than propofol (5, 30). However, significant fluctuations can still occur, particularly in children with congenital heart disease (32, 46) or during co-administration with opioids (31).

Other potential adverse events associated with remimazolam include:

1. PONV: Its incidence is multifactorial, and any potential advantage over inhaled anesthetics requires confirmation through high-quality pediatric data.
2. ED: Remimazolam may offer advantages over sevoflurane in preventing ED, but further research is needed to confirm this and optimize its application strategy.
3. Other reported events include dizziness, headache, somnolence, nausea, and vomiting. Rare instances of paradoxical agitation or excitement have also been observed; Kimoto et al. (31) reported agitation in approximately 1.9% and somnolence in 0.7% of children in their retrospective cohort. Furthermore, extremely rare case reports of severe allergic reactions, including anaphylaxis and circulatory collapse, have emerged (53, 54), highlighting the need for clinicians to maintain vigilance for these potentially life-threatening adverse events.
4. Formulation-specific anaphylaxis risk (Dextran 40):

A critical safety distinction exists between formulations. Remimazolam besylate (e.g., BYFAVO®) contains Dextran 40, a solubilizing excipient associated with severe anaphylactic reactions. This risk is specific to the excipient, not the remimazolam molecule itself. In contrast, remimazolam tosylate formulations (available in parts of Asia) do not contain Dextran 40. Clinicians must identify the specific formulation in use and screen for history of hypersensitivity to dextran-containing volume expanders.

### 4.2 Neurodevelopmental safety: a major, unresolved question and ethical imperative

The issue of long-term neurodevelopmental safety represents the most significant unresolved concern a core ethical consideration that must be rigorously addressed before the widespread adoption of any centrally acting anesthetic agent, including remimazolam, in young children, particularly those under 2 years of age during critical periods of brain development (55). The GABAA receptor system plays a crucial role in normal brain maturation. Consequently, the long-term impact of exogenous GABAA receptor agonists on the developing brain remains a subject of intense focus, concern, and ongoing debate within the pediatric anesthesia community (56–58).

Extensive preclinical research, primarily in rodent models, has demonstrated that various anesthetic agents, under specific exposure conditions (e.g., prolonged or repeated exposures), can induce neuronal apoptosis, alter synaptogenesis, and lead to long-term deficits in learning and behavior (58). However, the translation of these findings from rodent models to human children is complex and uncertain. The effects of GABAA agonists appear to be dependent on dose, duration, frequency of exposure, and the developmental stage at the time of exposure: high-dose, prolonged, or repeated exposures are more likely associated with neurotoxic potential, whereas low-dose, brief, or single exposures might have minimal impact or could even exert neuroprotective effects under specific pathological conditions like ischemia (59).

Research specifically investigating the neurodevelopmental safety of remimazolam itself is extremely limited, and the available results are inconsistent or even contradictory: One study in mice by Lu et al. (59) suggested that remimazolam might impair the consolidation of fear extinction memory, potentially by enhancing specific inhibitory pathways, raising concerns about interference with crucial learning and adaptation mechanisms. This finding warrants significant caution. Conversely, a study by Shi et al. (60) in a rat model of brain ischemia found that remimazolam attenuated neuronal injury, possibly involving antioxidant and anti-apoptotic pathways; however, this finding in a disease model absolutely cannot be interpreted as evidence of safety or benefit for the normally developing brain. Another study in young rats (61) reported cognitive effects, but the underlying mechanisms and clinical relevance remain to be elucidated.

Critically, clinical data on long-term neurodevelopmental outcomes in children exposed to remimazolam are completely absent. The lack of validated pediatric PK/PD models and TCI systems (16, 28, 30) further complicates precise control over brain drug exposure. Smooth short-term recovery must not be used to dismiss potential long-term neurodevelopmental sequelae; addressing this gap through well-designed longitudinal studies remains an ethical imperative.

## 5 Clinical positioning and individualized selection of remimazolam in pediatric day surgery anesthesia: balancing potential and risks

Before discussing specific clinical scenarios, it is critical to emphasize the regulatory context. To our knowledge, remimazolam is currently approved by major regulatory bodies (e.g., FDA, EMA) primarily for adult indications. Consequently, pediatric use constitutes off-label administration in most jurisdictions. This status necessitates enhanced clinical governance, including specific informed consent discussions with guardians regarding the rationale and alternatives, as well as adherence to institutional protocols for off-label medication use.

The following discussion on clinical positioning builds upon the evidence critically reviewed earlier. Table 1 provides a consolidated overview of these key studies, their findings, and identified limitations.

The research gathered in Table 1, which constitutes a significant portion of the existing evidence concerning pediatric remimazolam, offers crucial preliminary insights but exhibits shared methodological shortcomings that hinder their validity and applicability. Commonly noted limitations involve small sample sizes, age restrictions (particularly the omission of infants under 2 years), and designs that are susceptible to bias (such as single-center and retrospective studies). There is also considerable variability in dosing, methods of outcome evaluation (both subjective and objective), terminology (for instance, definitions of emergence delirium), and the control of confounding factors. Blinding is frequently insufficient, especially in comparative studies, which increases the risk of performance bias, while the statistical power of some studies is limited. Importantly, vital domains, including pediatric-specific pharmacokinetic/pharmacodynamic modeling and evaluations of long-term neurodevelopmental safety, are largely absent. Thus, it is critical to recognize that referencing these studies stems from the current deficiency of more robust evidence. The interpretations of their results should be approached with significant caution, highlighting the urgent necessity for future research of higher quality and increased rigor as discussed further on.

### 5.1 Regulatory status and off-label considerations

Remimazolam is currently FDA- and EMA-approved only for adults. Consequently, all pediatric use is off-label. This status necessitates enhanced clinical governance:

1. Informed consent: Clinicians must explicitly disclose the off-label status to guardians, documenting the rationale (e.g., specific benefit over propofol) and discussing alternatives.
2. Institutional oversight: Usage should ideally occur within institutional quality improvement registries to monitor adverse events, given the paucity of large-scale safety data.

### 5.2 Evidence-based clinical scenarios

Based on its theoretical pharmacological profile and the limited available evidence, remimazolam’s theoretical value in pediatric day surgery anesthesia may be most relevant in specific clinical scenarios: (1) Scenarios prioritizing hemodynamic stability: For children with limited cardiovascular reserve (38, 46), poor tolerance to hypotension, or undergoing procedures where strict blood pressure control is critical, remimazolam’s possible gentler cardiovascular impact compared to propofol, as suggested by preliminary studies, could represent a theoretical advantage. However, this remains to be confirmed in larger, well-designed trials. (2) Scenarios focusing on recovery quality and rapid turnover: In children at high risk for ED (e.g., preschoolers, those with a history of preoperative anxiety or behavioral issues) or undergoing surgeries associated with a high incidence of ED (e.g., ENT or ophthalmologic procedures), remimazolam’s demonstrated potential to reduce ED compared to sevoflurane (7, 8, 48) is clinically attractive. This could improve the experience for both the child and family and reduce the need for interventions in the PACU. Furthermore, its theoretical rapid and relatively predictable recovery profile (5, 46) might contribute to optimizing surgical throughput and PACU workflow efficiency, particularly valuable in high-volume ambulatory surgery settings. (3) Improving the induction experience: The virtual absence of injection pain offers a clear and significant advantage over propofol, enhancing patient comfort and cooperation during IV induction. (4) Procedural sedation: Its characteristics of rapid onset, titratability, and relatively favorable safety profile make it a potentially suitable agent for providing moderate to deep sedation during various non-surgical procedures, such as endoscopy (33, 44, 45), diagnostic imaging (e.g., MRI) (45), or cardiac catheterization (32).

### 5.3 Limitations and risk management

Despite potential advantages, remimazolam use is subject to significant limitations necessitating careful risk assessment:

1. Management of respiratory depression risk is paramount: This remains the single most critical safety consideration associated with remimazolam use. It mandates that anesthesia teams possess robust respiratory monitoring capabilities (with routine use of ETCO₂ monitoring being strongly recommended (51)) along with proficient skills and readily available equipment for managing the pediatric airway.
2. Paucity of robust pediatric evidence: The most significant current constraint is the severe lack, or even complete absence, of high-quality clinical evidence, particularly in crucial pediatric subpopulations such as infants under 2 years of age and children with significant comorbidities (e.g., cardiac, hepatic, renal disease, obesity).
3. Cost Considerations and Health Economic Uncertainty.

Remimazolam’s acquisition cost is substantially higher than traditional agents. In the United States, a single 20 mg vial costs approximately $30–50 USD compared to less than $5 USD for equivalent propofol doses, raising important questions about cost-effectiveness in resource-constrained settings.

Theoretical cost-offset mechanisms include reduced PACU length of stay, decreased interventions for adverse events (emergence delirium, PONV), and improved operating room throughput. However, rigorous pharmacoeconomic analyses supporting these benefits remain absent. Critically, modest PACU time reductions (1–5 min) observed in cited studies may not translate to meaningful savings in typical pediatric ambulatory settings with fixed staffing and bed allocation. Cost-effectiveness likely varies across healthcare settings and patient populations—facilities with high baseline ED or PONV rates might see greater value than high-volume centers with already efficient turnover.

No published studies have conducted comprehensive health economic evaluations comparing remimazolam-based anesthesia against standard care in pediatric populations. Until such evidence emerges, clinicians must weigh potential clinical advantages against demonstrable cost increases, particularly in resource-limited settings. Future research should prioritize pragmatic, multicenter health economic evaluations stratified by surgical type, patient risk factors, and institutional setting.

1. Lack of intrinsic analgesic effect: This necessitates the integration of remimazolam into a multimodal analgesic strategy.
2. Challenges in BIS monitoring interpretation: The tendency for remimazolam to produce relatively high BIS values despite adequate clinical anesthesia (39) requires clinicians to rely more heavily on clinical signs, potentially interpret raw EEG data, or adapt interpretation thresholds, adding complexity to anesthetic depth management.

### 5.4 Clinical decision-making framework

The following clinical decision-making framework is built directly upon the critical appraisal of evidence detailed in Table 1. The recommendations are designed to translate the existing research findings, and more importantly their limitations, into practical clinical guidance.

Specifically, the “Scenarios Favoring Caution or Avoidance” are derived from the significant evidence gaps identified, such as the virtual absence of high-quality data in vulnerable populations (e.g., infants <2 years of age and children with severe comorbidities). Furthermore, the “Key Management Strategies” are direct, proactive responses to the challenges and risks observed in the available studies. For instance, the emphasis on strict respiratory and hemodynamic monitoring addresses the volatility reported in studies by Kimoto et al. (31) and Shimizu et al. (32), while the mandatory co-administration of analgesics is a direct consequence of remimazolam’s lack of intrinsic analgesic properties, a fact underlying all reviewed studies. This framework should therefore be applied cautiously, always prioritizing patient safety in light of these evidence gaps (Table 2).

**Table 2:** Clinical decision-making framework for remimazolam use in pediatric day surgery anesthesia.

Consideration factor	Scenarios favoring remimazolam use	Scenarios favoring caution or avoidance	Key management strategies
Patient characteristics	ASA physical status I-II Age >3 years Absence of severe comorbidities Significant preoperative anxiety History of emergence delirium (ED) following previous sevoflurane anesthesia.	Infants and young children <2 years of age ASA physical status III-IV Severe cardiopulmonary dysfunction Neuromuscular disorders Known or suspected abnormal Carboxylesterase 1 (CES1) enzyme activity History of epilepsy Chronic benzodiazepine users.	Thorough assessment of the child’s overall condition Develop individualized dosing regimens Enhanced monitoring of vital signs and depth of anesthesia.
Surgical type/duration	Short, elective procedures Procedures with high risk of ED (e.g., ENT, ophthalmic surgery), where preliminary evidence suggests remimazolam can reduce its incidence Procedural sedation requiring extended duration (e.g., MRI, endoscopy).	Complex surgeries with anticipated long duration or significant trauma Procedures involving intense painful stimuli Requirement for a comprehensive multimodal analgesia plan.	Integrate multimodal analgesia strategies (e.g., prioritize regional anesthesia, intraoperative opioids) Ensure adequate analgesia.
Anesthetic goals	Desire for rapid and smooth anesthetic induction Avoidance of injection pain Potential for rapid recovery, while being mindful of inter-individual variability and potential delays compared to adults Goal of reducing ED.	Need for precise titration of anesthetic depth (e.g., during intraoperative neurophysiological monitoring [IONM] such as MEPs in neurosurgery) High reliance on the accuracy of Bispectral Index (BIS) values.	Assess depth of anesthesia by integrating clinical signs (e.g., vital signs, spontaneous respiration, movement) Close monitoring of end-tidal carbon dioxide (ETCO2) Evaluate compatibility with MEP monitoring (if applicable).
Team/Equipment	Team proficient in pediatric airway management skills Availability of ETCO2 monitoring equipment Post-Anesthesia Care Unit (PACU) equipped for extended monitoring Readily available antagonist (flumazenil).	Lack of ETCO2 monitoring equipment Limited team experience in pediatric airway management Limited PACU monitoring capacity or staffing.	Ensure availability of adequate monitoring devices and management capabilities Develop contingency plans (e.g., difficult airway, respiratory depression).
Drug characteristics	Theoretically short context-sensitive half-time (CSHT) Availability of a specific antagonist (flumazenil) Minimal pain on intravenous injection More stable hemodynamics during induction compared to propofol, but significant fluctuations can still occur intraoperatively.	Dose-dependent respiratory depression (key risk) Lack of analgesic effect Potential difficulty or delay in interpreting BIS values Insufficient pediatric pharmacokinetic/pharmacodynamic (PK/PD) data Relatively high drug cost Risk of anaphylaxis (formulation-specific, e.g., Dextran 40).	Strict monitoring of respiratory function (rate, depth, ETCO2, SpO2) Mandatory co-administration of analgesics Assess depth of anesthesia based on a combination of clinical signs Careful evaluation of cost-effectiveness Screen for dextran hypersensitivity; Identify specific formulation (besylate vs. tosylate).
Evidence base	Preliminary studies in healthy children suggest: Potential reduction in ED Faster emergence More stable hemodynamics.	Lack of high-quality evidence in key populations (e.g., infants < 2 years, children with significant comorbidities) Off-label pediatric use (informed consent required, institutional policies variable) Unknown long-term safety data.	Prudent application based on current evidence Acknowledge the limitations of current evidence Pediatric pharmacokinetic/pharmacodynamic (PK/PD) models require further refinement Encourage participation in or attention to high-quality clinical research.

All pediatric use of remimazolam currently constitutes off-label administration in most regulatory jurisdictions (see Section 5.0). PACU, Post-anesthesia care unit; PK/PD, Pharmacokinetic/Pharmacodynamic; ED, Emergence delirium; PONV, Postoperative nausea and vomiting; ASA, American Society of Anesthesiologists physical status classification; ETCO₂, End-Tidal Carbon Dioxide; BIS, Bispectral Index; TCI, Target-controlled infusion; NSAIDs, Nonsteroidal anti-inflammatory drugs; RCTs, Randomized controlled trials.

### 5.5 Complementary positioning: a supplement, not a substitute

Based on the current evidence base, remimazolam should not be viewed as a universal replacement for established core anesthetic agents like propofol or sevoflurane. Instead, it is more appropriately positioned as a supplementary tool within the anesthesiologist’s armamentarium, possessing potential advantages in specific, carefully selected clinical scenarios. Its role should also be considered in relation to other agents. For instance, compared to dexmedetomidine, an *α*₂-adrenergic agonist providing unique ‘cooperative’ sedation, adjunctive analgesia, and sympatholysis with typically less respiratory depression (62–64), remimazolam generally offers faster onset and recovery. A review by Scheckenbach et al. (35) explored optimizing the use of both agents in pediatric sedation, but high-quality head-to-head trials directly comparing their efficacy and safety in specific pediatric contexts are currently lacking. Furthermore, the rational use of remimazolam should be integrated within the broader frameworks of multimodal analgesia and Enhanced Recovery After Surgery (ERAS) principles. Its application should be actively combined with regional anesthesia techniques (42) and non-opioid analgesics to minimize reliance on opioids and their associated side effects.

## 6 Future research priorities

To bridge current knowledge gaps, future research must focus on:

1. Vulnerable populations: Prioritizing PK/PD studies in infants <2 years and children with significant comorbidities (1, 17).
2. Methodology: Conducting multicenter, active-comparator RCTs incorporating patient-reported outcome measures (PROMs) (65).
3. Precision anesthesia: Developing and validating pediatric TCI systems and closed-loop delivery (16, 28, 39).
4. Long-term safety: Establishing large-scale cohorts to assess neurodevelopmental outcomes, building on methodologies from the GAS (56) and PANDA (57) trials.
5. Health economics: Performing rigorous cost-effectiveness analyses to justify the higher acquisition cost (66).

## 7 Conclusion: potential yet unproven, caution remains paramount

Remimazolam, with its unique pharmacological properties and esterase-mediated metabolism, may offer theoretical promise for addressing key challenges in pediatric day surgery anesthesia, particularly regarding the goals of achieving more precise anesthetic control and predictable, rapid recovery (20, 27, 28). However, as this critical review indicates, current evidence provides only preliminary and often low-quality suggestions of potential clinical value. The high-quality clinical evidence required to support the widespread, safe, and effective application of remimazolam across the diverse pediatric population remains severely deficient at present. These include potentially exhibiting relatively better hemodynamic stability compared to propofol (5), offering a painless intravenous infusion experience (5, 33), and possibly being more effective than sevoflurane in reducing the incidence of ED (7, 8, 48). Crucially, the clinical application of remimazolam must always be predicated on a profound understanding and strict, proactive management of its inherent risks. Dose-dependent respiratory depression stands as the foremost and most critical safety concern requiring constant vigilance (16, 19, 51). Significant knowledge gaps persist, including the extreme scarcity of robust clinical data in key vulnerable populations (especially infants <2 years and children with comorbidities) (1, 12, 13, 17); the lack of reliable, validated pediatric PK/PD models and TCI systems (15, 16); the complete uncertainty surrounding long-term neurodevelopmental safety (55, 58); and the absence of comprehensive cost-effectiveness analyses (66).

In conclusion, remimazolam represents a promising but currently immature addition to the pediatric anesthesia armamentarium. It is certainly not a “panacea” or a simple substitute for well-established existing agents. The current evidence base is characterized by small sample sizes, methodological limitations, and critical population gaps. Only through continued, rigorous, high-quality research designed to systematically fill the critical evidence gaps will it be possible to determine if and how remimazolam can ultimately fulfill its potential to play a beneficial and unique complementary role in shaping safer, more efficient, and truly individualized paradigms for pediatric day surgery anesthesia. Until then, a cautious and evidence-informed approach to its use is imperative.
